# Defining ethical challenge(s) in healthcare research: a rapid review

**DOI:** 10.1186/s12910-021-00700-9

**Published:** 2021-09-29

**Authors:** Guy Schofield, Mariana Dittborn, Lucy Ellen Selman, Richard Huxtable

**Affiliations:** 1 Centre for Ethics in Medicine, Population Health Sciences, Bristol Medical School, University of Bristol, Bristol, BS8 2PS UK; 2 Paediatric Bioethics Centre, Great Ormond Street Hospital, London, WC1N 3JH UK; 3 Palliative and End of Life Care Research Group, Population Health Sciences, Bristol Medical School, University of Bristol, Bristol, BS8 2PS UK

**Keywords:** Ethical challenges, Rapid review, Empirical bioethics, Moral dilemmas

## Abstract

**Background:**

Despite its ubiquity in academic research, the phrase ‘ethical challenge(s)’ appears to lack an agreed definition. A lack of a definition risks introducing confusion or avoidable bias. Conceptual clarity is a key component of research, both theoretical and empirical. Using a rapid review methodology, we sought to review definitions of ‘ethical challenge(s)’ and closely related terms as used in current healthcare research literature.

**Methods:**

Rapid review to identify peer-reviewed reports examining ‘ethical challenge(s)’ in any context, extracting data on definitions of ‘ethical challenge(s)’ in use, and synonymous use of closely related terms in the general manuscript text. Data were analysed using content analysis. Four databases (MEDLINE, Philosopher’s Index, EMBASE, CINAHL) were searched from April 2016 to April 2021.

**Results:**

393 records were screened, with 72 studies eligible and included: 53 empirical studies, 17 structured reviews and 2 review protocols. 12/72 (17%) contained an explicit definition of ‘ethical challenge(s), two of which were shared, resulting in 11 unique definitions. Within these 11 definitions, four approaches were identified: definition through concepts; reference to moral conflict, moral uncertainty or difficult choices; definition by participants; and challenges linked to emotional or moral distress. Each definition contained one or more of these approaches, but none contained all four. 68/72 (94%) included studies used terms closely related to synonymously refer to ‘ethical challenge(s)’ within their manuscript text, with 32 different terms identified and between one and eight different terms mentioned per study.

**Conclusions:**

Only 12/72 studies contained an explicit definition of ‘ethical challenge(s)’, with significant variety in scope and complexity. This variation risks confusion and biasing data analysis and results, reducing confidence in research findings. Further work on establishing acceptable definitional content is needed to inform future bioethics research.

## Background

Methodological rigour within research is a cornerstone in the production of high-quality findings and recommendations. Across the range of empirical methodologies, a broad collection of protocol development tools, methodology guidelines, and reporting guidelines have been developed and evidence of their use is increasingly required by journals [1–6]. Within both empirical bioethics and descriptive ethics, there has been an accompanying increase in the acknowledgment of the importance of methodological rigour in the empirical elements, including within the recent consensus statement on quality standards in empirical bioethics research by Ives et al. [7–9]. Aligned with this aim for rigour, definitional clarity of key terms used within a research project is a component of research quality [10, 11]. Improving the quality of empirical bioethics is also itself an ethical imperative [9].

We recently conducted a systematic review examining ‘ethical challenges’ as reported by specialist palliative care practitioners [12]. Our review, alongside our initial scoping search findings and reading of the literature, suggested that, although many authors use the term ‘ethical challenge(s)’ in empirical ethics research, there appeared to be no commonly described or accepted definition. Furthermore, papers retrieved rarely defined ‘ethical challenge(s)’ explicitly*,* which has also been noted by other researchers examining other topic areas [13–15]. Our review further suggested that authors frequently use terms closely related to ‘ethical challenge(s)’—such as ‘moral dilemmas’ or ‘ethical issues’—interchangeably with ‘ethical challenge(s)’ throughout manuscripts, rather than staying with the original term. Research shows that non-philosophers may understand these related terms in heterogeneous ways which may additionally affect understanding of texts across different readerships [16, 17].

Without a clear definition of an ethical challenge, each researcher must use individual judgement to ascertain whether they have identified an instance of one within their dataset. This potentially generates an unnecessary source of bias, particularly if multiple researchers are involved in data collection, extraction, or analysis. This risks generating misleading ethical analyses, evaluations, or recommendations. Additionally, and more broadly, if primary studies do not define the term, then work based on these—such as systematic reviews of individual studies or those undertaking secondary data analysis—may unknowingly compare different phenomena without a mechanism for mitigating the effects this introduces.

In the hope of prompting a debate on this topic, we therefore undertook a rapid review, which aimed to explore existing definitions of “ethical challenge(s)” and the use of other closely related terms within recent empirical healthcare ethics literature.

## Methods

We conducted a rapid review examining the usage of the term ‘ethical challenge(s)’ over the last 5 years in published research articles, in order to identify and summarise if, and how, the term was defined. As a secondary aim, we examined authors’ uses of closely related alternative terms within the included article texts separate to their use within any explicit definitions that may be present.

Rapid reviews use abridged systematic review methodology to understand the evidence base on a particular topic in a time and resource efficient manner [18–22]. Comparative reviews of topics in which both a rapid review and a systematic review had been undertaken demonstrated that the overall conclusions were similar, although rapid reviews were less likely to contain social and economic data, and systematic reviews contained more detailed recommendations [18–20, 23, 24]. The Cochrane Rapid Review Methods Group has recently released interim methodological guidelines for undertaking rapid reviews [6], advising authors to describe where their protocol deviates from a systematic review and detail any biases that these deviations may introduce [18, 19, 21]. We have followed the Cochrane recommended methodology [6]. A rapid review reporting guideline is currently under development [25] and this review is therefore reported based on the PRISMA 2020 statement for systematic reviews, with justifications provided where our approach deviated [26].

Prospective review protocol registration on the PROSPERO database is the current gold standard, but, at the time of writing, PROSPERO does not accept records for rapid reviews [27]. The protocol was therefore not published in advance.

### Eligibility criteria

The inclusion and exclusion criteria are summarised in Table 1. We used Strech et al.’s Methodology, Issues, Participants (MIP) structure for our eligibility criteria, which is recommended for systematic reviews in ‘empirical bioethics’ [28]. The criteria reflect three assumptions. First, that the inclusion of ‘ethical challenge(s)’ in the title would increase the likelihood that this was the authors’ preferred term for the concept under investigation, and therefore increase the probability of a definition being provided. Second, that studies aiming to describe empirical data and identify ethical challenges in real-world contexts are most likely to contain a definition to guide researchers in identifying these challenges as they collect and analyse data. Third, that structured reviews of studies of ethical challenges are likely to include a definition to allow researchers to reliably recognise an ethical challenge in retrieved records. We used a 5-year timeframe as a date restriction. This reflected a balance between adequately covering recent use of the term and time and resource restrictions of the rapid review.

**Table 1:** Inclusion and exclusion criteria

	Inclusion criteria	Exclusion criteria
Types of participants	Any participants	No study will be excluded based on participant characteristics
Issues	Studies examining ‘ethical challenge(s)’ in any healthcare context	Studies not reporting research in a healthcare context
Methodologies	Qualitative studies, mixed methods and quantitative studies, systematic reviews, structured but non-systemic reviews (narrative syntheses, rapid reviews, scoping reviews and other records with a described protocol that could be independently followed.) or their published protocols	These may include expert opinion, bioethical argument pieces or case studies and analysis.Expert reviews on topics with no formal structure or published protocol details
Timeframe	Five years. Publications indexed between 01/04/2016 and 31/03/2021	Indexed outside of this timeframe
Type of publications	Reports that contain the phrase ‘ethical challenge(s)’ in the title Peer-reviewed journal publications of empirical research or structured reviews published in English	Where no full text is available through the university subscription, study authors will be contacted for full text. If there is no response within two weeks, the study will be excluded The following will also be excluded: Conference abstracts Editorials, letters, or comment/opinion pieces Book sections

### Information sources

The search strategy was as follows:

‘ethical challenge’.ti OR ‘ethical challenges’.ti.

We searched Medline (Ovid interface), Philosopher’s Index (OVID interface), EMBASE (OVID interface), and CINAHL (Cumulative Index to Nursing and Allied Health Literature, EBSCO interface) for studies indexed over a five-year period between April 2016 and April 2021. These resources cover the breadth of healthcare research. Including Philosopher’s Index increased coverage of the bioethics literature. We did not search the grey literature [6]. The search strategy was tested by successfully retrieving three sentinel studies known to the research team.

### Study selection

Retrieved studies were imported into Endnote X9.2 [29]. Records unavailable through institutional subscriptions were requested from corresponding authors. If unavailable 14 days after the request, the record was excluded. A random sample of 20% of records were dual screened at the title/abstract level by GS/MD. After discussion, the remainder were screened by GS. At full-text screening, a further 20% were dual screened by GS/MD and, again after discussion, the remaining studies were screened by GS.

### Data extraction and analysis

Data extraction was undertaken using a pre-piloted form, with the first 5 records dually extracted by GS and MD. Data from the remaining included studies was then extracted by GS, with correctness and completeness checked by MD. We collected data on date of publication, authors, journal, country (for primary studies), methodology, definition of ‘ethical challenge(s)’ (present (yes/no)) and (where offered) the definition provided, and any closely related terms used, with counts of all terms used in each article. For closely related terms, data was extracted from the authors’ text, but not from direct quotations from qualitative research. Where definitions of ‘ethical challenge(s)’ were offered and/or related terms were identified, these were categorised and counted following the principles of summative content analysis [30]. Summative content analysis combines both the quantitative counting of specific content or words/terms with latent content analysis to identify and categorise their meanings. We identified keywords (‘ethical challenge(s)’ and closely related terms) deployed by the authors of the included papers, both prior to and during data analysis, and analysed the retrieved definitions. This approach allowed for exploration of both the content of definitions and development of insights into the use of related terms.

### Risk of bias assessment

The focus of the rapid review was the definition of the term ‘ethical challenge(s)’ within retrieved records. We therefore did not undertake quality assessment for the included studies and reviews.

## Results

**Fig. 1:**
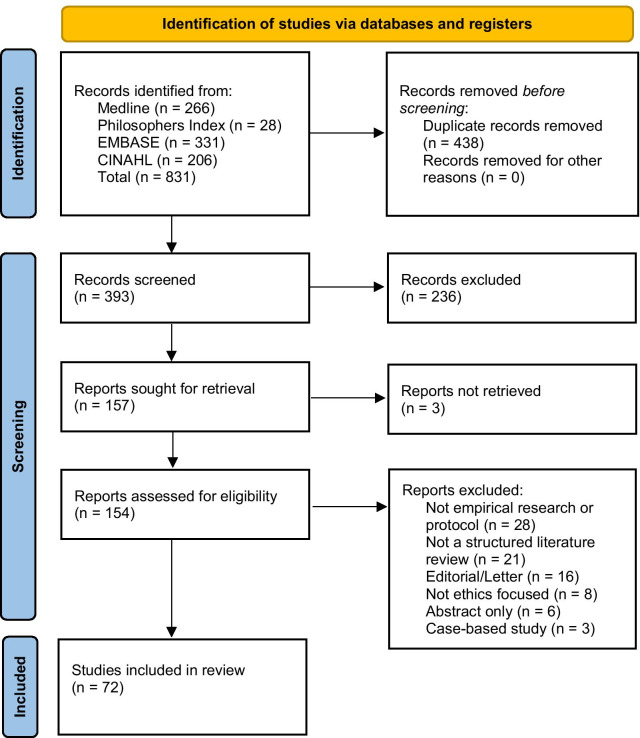
PRISMA flow diagram of record identification

831 records were retrieved, reduced to 393 after de-duplication. 238 records were excluded after reviewing the title and/or abstract. 157 records were identified for full text screening, with 3 unavailable [31–33]. 82 records were excluded at full text stage and 72 records were included for analysis. See Fig. 1 for the PRISMA flowchart.

### Record characteristics

Of the 72 included records, 53 were empirical studies [34–86], 10 non-systematic reviews [87–96], 7 systematic reviews [12–14, 97–100], 1 systematic review protocol [101], and 1 non-systematic review protocol [102]. Of the 53 empirical studies, 42 (79%) were qualitative studies [34–36, 38–44, 47, 48, 50–52, 54–58, 60, 62–67, 69, 71–77, 79–81, 83–86], 6 (12%) used a mixed methods approach [45, 46, 53, 59, 61, 68], and 5 (10%) were quantitative [37, 49, 70, 78, 82]. 7/56 empirical studies, all qualitative interview studies, recruited participants internationally with no specific location stated [40, 54, 55, 58, 60, 63, 73]. Of the remaining studies, all but one were single-country studies: Botswana [75], Canada [41, 65], China [57], Denmark [39, 43], Dominican Republic [44], Germany [51, 84], India [61], Iran [38, 46, 49, 68, 70–72, 78, 82, 98], Italy [45], Mexico [87], the Netherlands [76], New Zealand [47], Norway [42, 52, 56, 64, 80, 81, 83], Saudi Arabia [34–37], Tanzania [69, 74], Uganda [67], UK [86], and USA [50, 53, 59, 62, 66, 77, 79, 85, 85]. The remaining study was undertaken in both Sierra Leone and the UK [48]. See Table 2 for a summary.

**Table 2:** Included study details

References	Title	Published	Country	Research Methodology	Definition
Draper and Jenkins [48]	Ethical challenges experienced by UK military medical personnel deployed to Sierra Leone (operation GRITROCK) during the 2014–2015 Ebola outbreak: a qualitative study	2017	UK/Sierra Leone	Qualitative	Y
Forbes and Phillips [50]	Ethical Challenges Encountered by Clinical Trials Nurses: A Grounded Theory Study	2020	USA	Qualitative	Y
Hem et al. [14]	Ethical challenges when using coercion in mental healthcare: A systematic literature review	2018	n/a review	Systematic Review	Y
Heggestad et al. [13]	Ethical challenges in home-based care: A systematic literature review	2020	n/a review	Systematic Review	Y
Jakobsen and Sørlie [56]	Ethical challenges: Trust and leadership in dementia care	2016	Norway	Qualitative	Y
Jia et al. [57]	Nurses' ethical challenges caring for people with COVID-19: A qualitative study	2021	China	Qualitative	Y
Larkin et al. [66]	Ethical challenges experienced by clinical research nurses: A qualitative study	2019	USA	Qualitative	Y
Mlughu et al. [69]	Voluntary HIV Counseling and Testing Among Commercial Motorcyclist Youths: An Exploration of Ethical Challenges and Coping Mechanisms in Dar es Salaam	2020	Tanzania	Qualitative	Y
Saghafi et al. [98]	Examining the ethical challenges in managing elder abuse: a systematic review	2019	n/a review	Systematic Review	Y
Schofield et al. [101]	Real-world ethics in palliative care: protocol for a systematic review of the ethical challenges reported by specialist palliative care practitioners in their clinical practice	2019	n/a review	Systematic Review protocol	Y
Schofield et al. [12]	Real-world ethics in palliative care: A systematic review of the ethical challenges reported by specialist palliative care practitioners in their clinical practice	2021	n/a review	Systematic Review	Y
Storaker et al. [81]	From painful busyness to emotional immunization: Nurses' experiences of ethical challenges	2017	Norway	Qualitative	Y
Alahmad et al. [34]	Ethical challenges regarding the use of stem cells: interviews with researchers from Saudi Arabia	2020	Saudi Arabia	Qualitative	N
Alahmad et al. [35]	Ethical Challenges of Pediatric Cancer Care: Interviews With Nurses in Saudi Arabia	2020	Saudi Arabia	Qualitative	N
Alahmad et al. [37]	Ethical challenges in consent procedures involving pediatric cancer patients in Saudi Arabia: An exploratory survey	2021	Saudi Arabia	Qualitative	N
Alahmad et al. [36]	Ethical Challenges Related to the Novel Coronavirus (COVID-19) Outbreak: Interviews With Professionals From Saudi Arabia	2021	Saudi Arabia	Qualitative	N
Ayala-Yáñez et al. [87]	Violence against trainees: urgent ethical challenges for medical educators and academic leaders in perinatal medicine	2020	n/a Review	Non-systematic Review	N
Bijani and Mohammadi [38]	Ethical challenges of caring for burn patients: a qualitative study	2021	Iran	Qualitative	N
Binns et al. [88]	Ethical Challenges in Infant Feeding Research	2017	n/a Review	Non-systematic Review	N
Bladt et al. [39]	Empirical Investigation of Ethical Challenges Related to the Use of Biological Therapies	2020	Denmark	Qualitative	N
Boulanger et al. [40]	Developing and Implementing new TB Technologies: Key Informants' Perspectives on the Ethical Challenges	2020	International	Qualitative	N
Bourbonnais et al. [41]	Conditions and ethical challenges that could influence the implementation of technologies in nursing homes: A qualitative study	2019	Canada	Qualitative	N
Brodtkorb et al. [42]	Preserving dignity in end-of-life nursing home care: Some ethical challenges	2017	Norway	Qualitative	N
Bruun et al. [43]	Ethical challenges assessed in the clinical ethics Committee of Psychiatry in the region of Southern Denmark in 2010–2015: a qualitative content analyses	2018	Denmark	Qualitative	N
Canario Guzmán et al. [44]	Ethical challenges for international collaborative research partnerships in the context of the Zika outbreak in the Dominican Republic: a qualitative case study	2017	Dominican Republic	Qualitative	N
Carnevale et al. [45]	Correctional nursing in Liguria, Italy: examining the ethical challenges	2018	Italy	Mixed-methods	N
Cartolovni and Habek [89]	Guidelines for the management of the social and ethical challenges in brain death during pregnancy	2019	n/a review	Non-systematic Review	N
Delpasand et al. [46]	Ethical challenges in the relationship between the pharmacist and patient in Iran. International Journal of Human Rights in Healthcare	2020	Iran	Mixed Methods	N
Donnelly and Walker [47]	Enabling first and second year doctors to negotiate ethical challenges in end-of-life care: a qualitative study	2021	New Zealand	Qualitative	N
Ebrahimi and Ebrahimi [49]	Pediatric residents' and attending physicians' perspectives on the ethical challenges of end of life care in children	2018	Iran	Quantitative	N
Ewuoso et al. [100]	How do healthcare professionals respond to ethical challenges regarding information management? A review of empirical studies	2021	n/a review	Systematic Review	N
Forbes and Phillips [50]	Ethical Challenges Encountered by Clinical Trials Nurses: A Grounded Theory Study	2020	USA	Qualitative	N
Gagyor et al. [51]	Ethical challenges in primary care: a focus group study with general practitioners, nurses and informal caregivers	2019	Germany	Qualitative	N
Haugom et al. [52]	Ethical challenges of seclusion in psychiatric inpatient wards: a qualitative study of the experiences of Norwegian mental health professionals	2019	Norway	Qualitative	N
Hawking et al. [53]	"Can virtue be taught?": a content analysis of medical students' opinions of the professional and ethical challenges to their professional identity formation	2020	USA	Mixed-methods	N
Hofmann [90]	Informing about mammographic screening: Ethical challenges and suggested solutions	2020	n/a review	Non-systematic Review	N
Hunt et al. [91]	Ethical Challenges in the Provision of Mental Health Services for Children and Families During Disasters	2018	n/a review	Non-systematic Review	N
Hyder and Krubiner [54]	Ethical Challenges in Designing and Implementing Health Systems Research: Experiences from the Field	2016	International	Qualitative	N
Jackson et al. [55]	Trust and the ethical challenges in the use of whole genome sequencing for tuberculosis surveillance: a qualitative study of stakeholder perspectives	2019	International	Qualitative	N
Johnson and Parker [92]	Ethical challenges in pathogen sequencing: a systematic scoping review	2020	n/a review	Non-systematic Review	N
Kalkman et al. [58]	Stakeholders' views on the ethical challenges of pragmatic trials investigating pharmaceutical drugs	2016	International	Qualitative	N
Kasper et al. [59]	Perspectives and Solutions from Clinical Trainees and Mentors Regarding Ethical Challenges During Global Health Experiences	2020	USA	Mixed-methods	N
Kelley et al. [60]	Ethical challenges in research with orphans and vulnerable children: A qualitative study of researcher experiences	2016	International	Qualitative	N
Kemparaj et al. [61]	The Top 10 Ethical Challenges in Dental Practice in Indian Scenario	2015	India	Mixed-methods	N
Klitzman [62]	Unconventional combinations of prospective parents: ethical challenges faced by IVF providers	2017	USA	Qualitative	N
Komparic et al. [63]	A failure in solidarity: Ethical challenges in the development and implementation of new tuberculosis technologies	2019	International	Qualitative	N
Laholt et al. [64]	Ethical challenges experienced by public health nurses related to adolescents' use of visual technologies	2019	Norway	Qualitative	N
Laliberte et al. [65]	Ethical Challenges for Patient Access to Physical Therapy: Views of Staff Members from Three Publicly-Funded Outpatient Physical Therapy Departments	2017	Canada	Qualitative	N
Larkin et al.[66]	Ethical challenges experienced by clinical research nurses:: A qualitative study	2019	USA	Qualitative	N
MacDonald and Shemie [93]	Ethical Challenges and the Donation Physician Specialist: A Scoping Review	2017	n/a review	Scoping Review	N
Martins Pereira and Hernandez-Marrero [97]	Ethical challenges of outcome measurement in palliative care clinical practice: a systematic review of systematic reviews	2018	n/a review	Systematic Review	N
Mbalinda et al. [67]	Ethical challenges of the healthcare transition to adult antiretroviral therapy (ART) clinics for adolescents and young people with HIV in Uganda	2021	Uganda	Qualitative	N
Mehdipour Rabori et al. [68]	Nursing students' ethical challenges in the clinical settings: A mixed-methods study	2019	Iran	Mixed-methods	N
Moeini et al. [70]	Ethical challenges of obtaining informed consent from surgical patients	2020	Iran	Quantitative	N
Morley et al. [86]	Moral Distress and Austerity: An Avoidable Ethical Challenge in Healthcare	2019	UK	Qualitative	N
Naseri-Salahshour and Sajadi [71]	Ethical challenges of novice nurses in clinical practice: Iranian perspective	2020	Iran	Q ualitative	N
Naseri-Salahshour and Sajadi [72]	From Suffering to Indifference: Reaction of Novice Nurses to Ethical Challenges in First Year of Clinical Practice	2019	Iran	Qualitative	N
Nicholls et al. [73]	The ethical challenges raised in the design and conduct of pragmatic trials: An interview study with key stakeholders	2019	International	Qualitative	N
Pancras et al. [74]	Non-medical facilitators and barriers towards accessing haemodialysis services: an exploration of ethical challenges	2018	Tanzania	Qualitative	N
Sabone et al. [75]	Everyday ethical challenges of nurse-physician collaboration	2020	Botswana	Qualitative	N
Saigle and Racine [94]	Ethical challenges faced by healthcare professionals who care for suicidal patients: a scoping review	2018	n/a review	Non-systematic Review	N
Saigle et al. [95]	Identifying Gaps in Suicide Research: A Scoping Review of Ethical Challenges and Proposed Recommendations	2017	n/a review	Non-systematic review	N
Seekles et al. [76]	Inspectors' Ethical Challenges in Health Care Regulation: A Pilot Study	2017	Netherlands	Qualitative	N
Segal et al. [77]	County Jail or Psychiatric Hospital? Ethical Challenges in Correctional Mental Health Care	2018	USA	Qualitative	N
Shayestefar et al. [78]	Ethical challenges in pediatrics from the viewpoints of Iranian pediatric residents	2018	Iran	Quantitative	N
Sinow et al. [79]	How Anesthesiologists Experience and Negotiate Ethical Challenges from Drug Shortages	2020	USA	Qualitative	N
Slettebo et al. [80]	Conflicting rationales: leader's experienced ethical challenges in community health care for older people	2018	Norway	Qualitative	N
Solvoll et al. [117]	Ethical challenges in everyday work with adults with learning disabilities	2015	Norway	Qualitative	N
Sun et al. [102]	Ethical challenges related to assistive product access for older adults and adults living with a disability: a scoping review protocol	2017	n/a review	Scoping Review Protocol	N
Taebi et al. [82]	Ethical Challenges of Embryo Donation in Embryo Donors and Recipients	2018	Iran	Quantitative	N
Tonnessen et al. [83]	Ethical challenges related to next of kin—nursing staffs' perspective	2016	Norway	Qualitative	N
Ullrich et al. [84]	Ethical challenges in family caregivers of patients with advanced cancer—a qualitative study	2020	Germany	Qualitative	N
Verma et al. [85]	Ethical Challenges in Caring for Unrepresented Adults: A Qualitative Study of Key Stakeholders	2019	USA	Qualitative	N
West et al. [99]	Operationalising ethical challenges in dementia research-a systematic review of current evidence	2017	n/a review	Systematic review	N
Wilson et al. [96]	Ethical Challenges in Community-Based Participatory Research: A Scoping Review	2017	n/a review	Non-systematic Review	N

### Findings

12/72 (17%) of retrieved studies offered an explicit definition for ‘ethical challenge(s)’ [12–14, 48, 50, 56, 57, 66, 69, 81, 98, 101]. Definitions were more likely to be found in more recent publications, with 4/12 included studies published in 2016–2018 [14, 48, 56, 81], and 8/12 published in 2019–2021 [12, 13, 50, 57, 66, 69, 98, 101]. The included study locations were evenly distributed, matching the overall pattern of retrieved studies, with studies from high- [48, 50, 56, 66, 81], middle- [57, 98], and low-income settings [48, 69]. The identified studies included eight qualitative studies [48, 50, 56, 57, 66, 69, 81, 98], 3 systematic reviews [12–14], and 1 systematic review protocol [101]. Two of these records were the systematic review protocol and the report from our group, which accordingly contained the same definition [12, 101], leaving 11 unique definitions. Definitions of ‘ethical challenge(s)’ identified in included studies are provided in Table 3. Additionally, 68/72 (94%) reports used closely related terms synonymously in place of ‘ethical challenge(s)’ throughout their manuscript text, with between 1 and 8 different terms used within each report, and 32 different terms were identified. This occurred in both those reports that contained a definition and those that did not. See Table 4 for terms and frequencies.

**Table 3:** Details of studies that contained an explicit definition of ‘ethical challenges’

Study	Study title	Study design	Study field, location	Definition	Concepts	Study participants	Conflict and uncertainty	Emotional or moral distress
Draper and Jenkins [48]	Ethical challenges experienced by UK military medical personnel deployed to Sierra Leone (operation GRITROCK) during the 2014–2015 Ebola outbreak: a qualitative study	Semi-structured interviews	Sierra Leone/UK	‘A shared understanding of what was meant by an ethical challenge was established either during the interview or immediately before it commenced. We took as our working definition that adopted by Schwartz et al.: ‘situations where either the HCPs [health care professionals] knew what they felt was the right thing to do but were somehow prevented from enacting it, or where “doing the right thing” also caused harm’.’		✓	✓	
Forbes and Phillips [50]	Ethical Challenges Encountered by Clinical Trials Nurses: A Grounded Theory Study	Online real-time typing interviews	Oncology clinical trial nurses USA	Study authors allowed participants to define ethical challenge. 'What does the term 'ethically challenging' mean to you' Results are described using participants’ descriptions		✓		
Heggestad et al. [13]	Ethical challenges in home-based care: A systematic literature review	Systematic Review	Home based care n/a review	‘Here, we have chosen to define an ethical challenge as “when there is doubt or disagreement about what is right or wrong.”’ This quotation references a definition used in Lillemoen L, Pedersen R. Ethical challenges and how to develop ethics support in primary health care. Nursing Ethics. 2013;20(1):96–108 [15]			✓	
Hem et al. [14]	Ethical challenges when using coercion in mental healthcare: A systematic literature review	Systematic Review	Mental Healthcare n/a review	‘An ethical challenge occurs when one does not know how to behave and act in the best way, when one feels doubt or discomfort or when one is uncertain with respect to how one should interact in or react to the situation.’			✓	✓
Jakobsen and Sorlie [56]	Ethical challenges: Trust and leadership in dementia care	Semi-structured interviews	Dementia care nursing home Norway	‘The single question invites the informants to express themselves openly through their narratives. Hence, it is up to them to define the situations that are ethically difficult.’		✓		
Jia et al. [57]	Nurses' ethical challenges caring for people with COVID-19: A qualitative study	Semi-structured interviews	COVID-19 Units China	‘The expression “ethical challenges” mainly refers to ethical dilemmas and ethical conflicts as well as other scenarios where difficult choices have to be made. Ethical dilemmas are described as situations that cannot be solved; decisions made between two options may be morally plausible but are equally problematic due to the circumstances. Ethical conflicts, on the contrary, arise when one is aware of the necessity of proper actions but he or she may have trouble exercising these actions because of certain internal or external factors.’	✓		✓	
Larkin et al. [66]	Ethical challenges experienced by clinical research nurses: A qualitative study	Semi-structured interviews	Clinical research nurse practice USA	‘For this study, “ethical challenges” were defined broadly to encompass ethical dilemmas, ethical conflicts, and other ethical issues potentially leading to moral distress and moral residue.’	✓			✓
Mlughu et al. [69]	Voluntary HIV Counseling [sic] and Testing Among Commercial Motorcyclist Youths: An Exploration of Ethical Challenges and Coping Mechanisms in Dar es Salaam	Interviews & focus groups	Commercial motorcyclist youths Tanzania	‘In this context, ethical challenge refers to the situation whereby every alternative is morally wrong and still one has to make a choice’			✓	
Saghafi et al. [98]	Examining the ethical challenges in managing elder abuse: a systematic review	Systematic Review	Elder abuse n/a review	‘ethical conflicts and challenges emerge when two or several ethical values relevant to a particular situation necessitate conflicting measures’			✓	
Schofield et al. [101]	Real-world ethics in palliative care: protocol for a systematic review of the ethical challenges reported by specialist palliative care practitioners in their clinical practice	Systematic review protocol	Palliative care n/a review	‘The definition of ‘ethical challenges’ will be intentionally kept broad to capture the maximum number of examples. It includes but is not limited to terms such as ethical issues, moral challenges, moral dilemmas, values, good/bad, right/wrong. Ethical challenges can be labelled as such either by authors or participants.’	✓	✓		
Schofield et al. [12]	Real-world ethics in palliative care: A systematic review of the ethical challenges reported by specialist palliative care practitioners in their clinical practice	Systematic review	Palliative care n/a review	‘The definition of ‘ethical challenges’ will be intentionally kept broad to capture the maximum number of examples. It includes but is not limited to terms such as ethical issues, moral challenges, moral dilemmas, values, good/bad, right/wrong. Ethical challenges can be labelled as such either by authors or participants.’	✓	✓		
Storaker et al. [81]	From painful busyness to emotional immunization: Nurses' experiences of ethical challenges	Interview study	Hospital nurses Norway	‘In this article, ethical challenges refer to values that entail emotional and moral stress in healthcare personnel.’				✓

**Table 4:** Use of terms closely related to ‘ethical challenge’

Number of studies containing the term (total = 75)	Term
> 30	Ethical issues
20–30	Ethical concerns Ethical dilemmas
11–20	Ethical aspects Ethical conflicts Ethical considerations Ethical problems
6–10	Ethically challenging/demanding/difficult situations Ethical difficulties Moral challenges
3–5	Ethical dimensions Ethical questions Ethical tensions Moral dilemmas
1–2	Ethical complications Ethical components Ethical difficulties Ethical discussions Ethical disquiet Ethical elements Ethical factors Ethical obstacles Ethical struggles Ethical uncertainties Moral conflict Moral courage Moral considerations Moral issues Moral problems Moral question Morally relevant topics Moral situations

Those records that offered explicit definitions used four approaches: (1) definition through concepts [12, 57, 66]; (2) reference to moral conflict, moral uncertainty or difficult choices [13, 14, 48, 57, 69, 98]; (3) definition by study participants [12, 48, 50, 56]; or (4) challenges as linked to their ability to generate emotional or moral distress within healthcare practitioners [14, 14, 66, 81]. Each definition was associated with one or more of the identified elements, although none covered all four approaches. We describe these approaches below.

### Approach 1: definition through concepts

This approach involves primarily defining ‘ethical challenge(s)’ in terms of related concepts. All three definitions using this approach defined ‘ethical challenge(s)’ as a summative collection of related concepts, including ‘ethical dilemmas’, ‘moral dilemmas’, ‘moral challenges’, ‘ethical issues’, and ‘ethical conflicts’ [12, 57, 66], for example:

‘The expression “ethical challenges” mainly refers to ethical dilemmas and ethical conflicts as well as other scenarios where difficult choices have to be made’ [57] p34

Only one went on to define the other concepts they utilised, ‘ethical dilemmas’ and ‘ethical conflicts’:

‘Ethical dilemmas are described as situations that cannot be solved; decisions made between two options may be morally plausible but are equally problematic due to the circumstances. Ethical conflicts, on the contrary, arise when one is aware of the necessity of proper actions but he or she may have trouble exercising these actions because of certain internal or external factors.’ [57] p34

### Approach 2: moral conflict, moral uncertainty or difficult choices

This approach anchors an ethical challenge to the requirement for an agent to make a (difficult) choice in a situation where moral principles conflict, or there is moral uncertainty as to the ‘right’ way forward.

‘In this context, ethical challenge refers to the situation whereby every alternative is morally wrong and still one has to make a choice’ [69] p676

‘An ethical challenge occurs when one does not know how to behave and act in the best way…’ [14] p93

### Approach 3: definition by study participants

Four of the definitions involved research participants themselves defining something as an ‘ethical challenge’ [12, 48, 50, 56], with three studies explicitly stating that participants would lead this definitional work [48, 50, 56]. Draper & Jenkins offer a starting definition, adopted from Schwartz et al. [103] with which to prime participants, while Forbes and Phillips [50] and Jakobsen and Sørlie [56] left the definition fully with their participants (Table 3). Finally, Schofield et al. proposed a very broad definition (Table 3), alongside the specific statement that either participants or researchers could nominate something as an ‘ethical challenge’ [12].

### Approach 4: emotional or moral distress

This final approach was to tie ethical challenges to situations where participants feel ‘discomfort’, emotional distress or more specifically moral distress or moral residue [14, 66, 81]. Larkin et al. are clear that this distress must be tied to moral causes, but Hem et al. and Storaker et al. also refer more broadly to *‘discomfort’* [14] and *‘emotional stress’* [81] respectively. For example:

‘In this article, ethical challenges refer to values that entail emotional and moral stress in healthcare personnel.’ [81] p557

## Discussion

To the authors’ knowledge, this is the first rapid review to examine the use of the term ‘ethical challenge(s)’ in empirical healthcare research literature. Notably, only 12/72 (17%) of included studies published in the last 5 years contained a definition for ‘ethical challenge(s)’, despite this being the focus of the research being reported. The definitions identified were found in qualitative studies and systematic reviews and were evenly distributed geographically across high-, middle- and low-income settings. Definitions contained one or more of the identified approaches, although none contained elements from all four. Taken together, these findings suggest that a clear definition of ‘ethical challenge(s)’, and consistent use thereof, is currently lacking.

The four approaches indicate the diverse approaches to understanding ‘ethical challenge(s)’. Approaches 1 and 2 explore the concept from opposite viewpoints, with approach 1 looking from the conceptual perspective, through terms such as ‘dilemmas’ and ‘conflict’, and approach 2 from a participant perspective, specifically in those situations in which someone is trying to make a decision in circumstances where the preferred option is not possible or when they perceive there to be clash in values they feel are important. Within the concept-led definitions (approach 1), the use of a plurality of terms highlights a potential risk of bias, as different readers may interpret these differently. For example, some terms, such as ‘moral dilemma’, have relatively well understood specific meanings for some readers, particularly those with philosophical training [104–106]. The presence in the literature of specific and multiple meanings for some related terms highlights the importance of empirical studies providing a definition of these additional terms alongside their primary definition for ‘ethical challenge(s)’. This is more likely to be relevant where an a priori definition is used, but may be relevant to any prompting text for studies using a participant-led process, as in the study by Draper and Jenkins [48]. This clarity is important for both readers and future researchers who may undertake a secondary analysis of the data.

Approach 3 involves facilitating participants to nominate something as an ethical challenge [12, 48, 50, 56]. This speaks to an important question about who, in a research context, is permitted to define or describe the object of interest, in this case ‘ethical challenge(s)’. Restricting the identification of ‘ethical challenge(s)’ to researchers alone may introduce bias by excluding input from those without bioethical ‘expertise’, but with important lived experience of the context under investigation. There is evidence that although clinicians can be sensitive to major ethical dilemmas, they can be less sensitive to small everyday ethical elements in clinical practice, and that ethical awareness varies between individuals [107, 108]. Additionally, there is evidence in healthcare ethics research that patients and carers identify ethical challenges in situations that healthcare workers do not [109]. Therefore, relying entirely on a particular stakeholders’ perspectives (such as clinicians’) may risk missing important ethical challenges present in a scenario (assuming, of course, that we can settle what counts as an ‘ethical challenge(s)’).

In Approach 4, ethical challenges were linked to situations in which participants felt discomfort [14], emotional stress [81], moral distress or moral residue [66]. These concepts are themselves defined in quite varied ways (see, for example, definitions of ‘moral distress’ in a systematic review by Morley et al. [110]), potentially leading to additional conceptual confusion. Identifying triggers for moral distress is important, as high levels of moral distress are known to have negative impacts on work environments and lead to increased levels of compassion fatigue, increased staff turnover rates and poorer patient outcomes [110–112]. However, it is also possible that the requirement that, to be identified as an ethical challenge, the situation must invoke stress or distress might result in the under-identification of ethical challenges. We anticipate that many practitioners will daily manage multiple low-level ethical challenges, many of which will not generate moral distress or leave a moral residue. As such, the presence of moral distress may not be sufficient or even necessary in order to label a moral event an ‘ethical challenge’. However, the relationship between ‘ethical challenge(s)’ and moral distress is complex, and some might argue that the latter has an important relationship to the former. For example, moral distress, as conceived by Jameton and others [110, 113, 114], is linked to the after-effects of having to handle ethical challenge(s), so some researchers might view the generation of moral distress as relevant to identifying ethical challenges.

Although our review revealed these four approaches, the wider literature indicates there may be alternative approaches available. For example, other potential approaches would define ethical challenges as events that interact with moral principles, such as autonomy, beneficence, non-maleficence or justice, as proposed by Beauchamp and Childress [115], or as events in which those principles clash, for example as used by Klingler et al. in their research focusing on ethical issues in health surveillance [116]. However, these approaches were not seen amongst our included papers.

Returning to our included papers, the high rates of use of closely related terms within included manuscript texts may add to difficulties in understanding the exact object of interest if these terms are being used as synonyms for ‘ethical challenge(s)’. This may be particularly the case if terms used include those such as ‘moral dilemma’, which (as shown above) will have specific meanings for some readers. Interchangeable, undefined usage of these terms by study authors within study texts risks further exacerbating the problems caused by a lack of definitional clarity.

### Strengths and limitations

This rapid review is the first systematic attempt to describe the definitions of ‘ethical challenge(s)’ available within the recent published literature.

There are, however, five limitations to note. First, the review only includes results from the past 5 years, which inevitably means that older publications, which may have contained further definitions of ‘ethical challenge(s)’, were excluded. The focus on the previous 5 years does, however, allow for an assessment of the term’s use(s) within a reasonable period of time and was felt to be appropriate given the aims and resources available to this project.

Second, our three assumptions listed in the methodology section may have excluded some records that contained a relevant definition. However, these assumptions, and the resulting focus on two search terms, allowed for a balance between retrieved record numbers and team resources.

Third, the four databases searched were chosen for their focus on the healthcare ethics literature; we may therefore may have missed relevant usage in other fields or disciplines. Similarly, we did not search the grey literature, which might have excluded relevant research.

Fourth, for resource reasons, the assessment as to whether a related term was being used interchangeably in the text was undertaken by a single researcher (GS). This subjective assessment risks miscalculating both the number of interchangeable terms identified and the frequency counts.

Finally, we did not review the theoretical literature for conceptual definitions of ‘ethical challenge(s)’, hence the definitions we identified might not match completely conceptual understandings of the term. However, our review shows how the term is currently being used in the research literature. Indeed, if there are strong conceptual definitions within the theoretical literature, then it is clear that they are currently not reaching the researchers whose work was identified by our review.

## Conclusions

This review is the first, to our knowledge, to identify and describe definitions (and uses) of the widely-utilised concept of ‘ethical challenge(s)’ within healthcare research. Only 17% (12/72) of retrieved papers presented an explicit definition of ‘ethical challenge(s)’ before beginning to investigate this concept in context. The definitions found contained one or more of four identified approaches, with significant cross-reference to related terms and concepts which themselves have variation in their accepted meanings. We recommend that researchers define the phenomenon of interest—in this case, ‘ethical challenge(s)’—to help ensure clarity. This should either be a priori, or, if using an approach that includes participant participation in the generation of the definition, reporting their final working definition a posteriori. The choice of definition should be justified, including the decision as to whether to include participants in this process. Additionally, if a definition references other conceptual terms, then consideration should be given to defining these as well.

The results of this rapid review suggest that a common conceptual understanding of the term ‘ethical challenge(s)’ is lacking within empirical bioethical research and that there is a need for researchers in this area to consider what conceptual formulations might be most useful. Again, failure to use definitions of crucial research concepts within empirical bioethics research potentially generates confusion and avoidable bias within research outputs, risking misleading ethical analyses, evaluations, and resulting recommendations. We therefore hope this review will help stimulate debate amongst empirical bioethics researchers on possible definitional content for such a commonly used term and prompt further discussion and research. Additionally, given the central role of patient and public partnership and involvement in research, further thought should be given to who should be involved in nominating something as a challenge worthy of study.

Following on from this work, there would be value in conducting an empirical bioethical project combining a full systematic review of definitions of ‘ethical challenge(s)’ (and related terms) integrated with an exploration of the conceptual literature to generate recommendations for approaches towards the content of potential definitions, perhaps related to the identified approaches above. Such a project could also ask authors who currently use the term ‘ethical challenge(s)’ in their research how they conceptualise this. Furthermore, work to better understand the benefits of including study participants in the definition process is also important. Finally, whilst researchers should justify whatever approach they choose to take, there may be merit in examining whether anything is lost if studies lack a robust or agreed definition, or whether doing so affords a flexibility and openness that allows for a broader range of ethical challenges to be identified.

## Data Availability

All data is presented in this manuscript.
